# Long-Term Adherence to Antiretroviral Treatment and Program Drop-Out in a High-Risk Urban Setting in Sub-Saharan Africa: A Prospective Cohort Study

**DOI:** 10.1371/journal.pone.0013613

**Published:** 2010-10-25

**Authors:** Christian Unge, Björn Södergård, Gaetano Marrone, Anna Thorson, Abigael Lukhwaro, Jane Carter, Festus Ilako, Anna Mia Ekström

**Affiliations:** 1 Division of Global Health (IHCAR), Department of Public Health Sciences, Karolinska Institutet, Stockholm, Sweden; 2 African Medical and Research Foundation, Kenya Country Program, Nairobi, Kenya; University of Cape Town, South Africa

## Abstract

**Background:**

Seventy percent of urban populations in sub-Saharan Africa live in slums. Sustaining HIV patients in these high-risk and highly mobile settings is a major future challenge. This study seeks to assess program retention and to find determinants for low adherence to antiretroviral treatment (ART) and drop-out from an established HIV/ART program in Kibera, Nairobi, one of Africa's largest informal urban settlements.

**Methods and Findings:**

A prospective open cohort study of 800 patients was performed at the African Medical Research Foundation (AMREF) clinic in the Kibera slum. Adherence to ART and drop-out from the ART program were independent outcomes. Two different adherence measures were used: (1) “dose adherence” (the proportion of a prescribed dose taken over the past 4 days) and (2) “adherence index” (based on three adherence questions covering dosing, timing and special instructions). Drop-out from the program was calculated based on clinic appointment dates and number of prescribed doses, and a patient was defined as being lost to follow-up if over 90 days had expired since the last prescribed dose. More than one third of patients were non-adherent when all three aspects of adherence – dosing, timing and special instructions – were taken into account. Multivariate logistic regression revealed that not disclosing HIV status, having a low level of education, living below the poverty limit (US$ 2/day) and not having a treatment buddy were significant predictors for non-adherence. Additionally, one quarter of patients dropped out for more than 90 days after the last prescribed ART dose. Not having a treatment buddy was associated with increased risk for drop-out (hazard ratio 1.4, 95% CI = 1.0–1.9).

**Conclusion:**

These findings point to the dilemma of trying to sustain a growing number of people on life-long ART in conditions where prevailing stigma, poverty and food shortages threatens the long-term success of HIV treatment.

## Introduction

Sustaining life-long antiretroviral treatment (ART) in the rapidly growing urban slums of sub-Saharan Africa, where 70% of urban populations live, is a major future challenge [1]. Urban slums are poorly supplied with basic services for health, education, sewage systems, water and electricity [1]. In combination with high unemployment, sexual risk behavior, overcrowding and insecurity, urban slums have worse health indicators than rural areas including higher rates of alcohol consumption, smoking, drug use and communicable diseases such as HIV [2], [3]. Health planning in the slums is a major challenge due to high mobility, especially since there is very little information about the burden of disease in the slums due to insufficient registration [4].

Adherence to ART is crucial for treatment success among HIV patients [5], [6], [7], [8]. High levels of adherence is a prerequisite for maintained viral suppression [9], [10] and a lower risk of drug resistance [11], [12] in turn preventing premature morbidity and mortality [5], [13]. Low adherence is the second strongest determinant for disease deterioration and death after CD4 count [14]. Non-adherence to ART is a substantial challenge in resource-poor settings like urban slums, where increasing drug resistance is hard to combat using the limited treatment alternatives available.

The number of patients discontinuing, or dropping out of ART in sub-Saharan Africa is believed to be substantial [15], [16]. Rosen et al (2007) found that, on average, 40% of patients enrolled in sub-Saharan African ART programs had discontinued their treatment after two years [17].

Nairobi, Kenya, has roughly 100 urban slums and, according to UN-Habitat estimates, about half of Nairobi's three million inhabitants live in slums. Kibera, one of Africa's largest urban slums, is situated in the heart of Nairobi and estimated to have a population of between 600 000 and 2 million, depending on the season. Specific adherence studies in urban slums are few but our group concluded in a retrospective study that 27% of patients attending the African Medical and Research Foundation (AMREF) clinic in Kibera had an overall mean adherence below 95% and that 29% discontinued the ART program [18]. The probability of remaining in the program was 0.65 after 2 years [18].

As more patients are initiated on life-long ART, one of the major future challenges, apart from securing sustainable funding, lies in retaining patients in care and in sustaining adherence to ART [19]. Much has already been done to achieve an understanding of the barriers and facilitators to ART adherence [17], [20], [21] but context-specific knowledge regarding urban slums is lacking [20], [22]. The objective of this study was to find determinants for low adherence to ART and program drop-out in a resource-poor, urban, sub-Saharan African setting.

## Methods

### Setting and population

Kenya has 38.3 million inhabitants and 1.6–1.9 million HIV-infected patients. At the end of 2008, WHO, UNAIDS and UNICEF estimated that approximately 44% of the people in need were receiving ART in sub-Saharan Africa. In Kenya, 308 000 adults were receiving ART at the end of 2009, corresponding to a coverage of 70.4% of those in need having been initiated on ART.

This study was conducted at the AMREF clinic in the Kibera slum, Nairobi, Kenya. Most people in Kibera live in small houses made of mud and corrugated metal with almost no access to electricity or sewerage. The majority lives off petty trade or casual, day-to-day labor. The slum houses people of about 40 different ethnic backgrounds from all over Kenya. The population is constantly changing since people are highly mobile. A vast number of health care providers, both national and international, offer different services for Kibera inhabitants but there is no, or very little, coordination between these providers. The AMREF clinic in Kibera offers preventive, diagnostic and basic health care, as well as care services for women and children. One medical officer, 3 clinical officers, 14 nurses, 2 nutritionists, 2 pharmacists and 8 community health care workers run the clinic. As the first ART provider in Kibera, the centre has offered free treatment for opportunistic infections and ART care and support to HIV-infected Kibera residents since February 2003. A treatment buddy (i.e. a friend or family member, known to the clinic, helping the patient taking ART) is not requested but patients are advised to have one. A nutrition program provides fortified flour to HIV-infected adults and patients with TB if they have a body mass index below 18.4, and to all HIV-infected children.

### ART regimens

Patients who present with WHO clinical stage 3 or 4, or, with a CD4-count of <250 cells/mm^3^ are eligible for ART at the AMREF clinic, according to Ministry of Health guidelines. Patients' CD4 cells, clinical status and, when there are signs of treatment failure, viral load are routinely monitored by the AMREF staff. Under normal conditions, patients collect their ART from the AMREF pharmacy every 30 days. First-line ART-regimes at the AMREF clinic include stavudine, lamivudine, and nevirapine/efavirez. Second-line regimes include zidovudine, abacavir, didanosine, ritonavir-boosted lopinavir (Kaletra) and tenofovir. By March 2010, the AMREF clinic in the Kibera slum had initiated 1 792 patients on ART.

### Data collection and study design

A prospective open cohort study was conducted. All consecutive patients, HIV-positive, ≥18 years of age, visiting the clinic during the study period (9 September, 2007–20 March 2010) and either on ART or in the process of starting ART were eligible for the study. Local clinical officers (CO) and a research assistant at the clinic were trained by the first author (CU) before study initiation. The COs, in collaboration with the attendant in charge, identified patients eligible for the study. One research assistant (AL) performed all baseline interviews with those patients who gave their informed consent to participate after double-checking their eligibility.

The baseline and follow-up questionnaires were developed in close collaboration with the AMREF staff, piloted on two occasions and adjusted several times after feedback from staff at the clinic with different professional backgrounds. The questionnaires were based on a modified Adult Aids Clinical Trials Group adherence questionnaire, (AACTG questionnaire) [23].

The baseline questionnaire consisted of 68 closed questions covering the following socio-demographic factors: age, gender, ethnicity, religion, civil status, number of children, level of education, income, work status, living arrangements and alcohol/drug consumption. The patients were also asked about disclosure of HIV status and social support. One question about adherence to ART was asked: “When was the last time you missed taking any of your medications? Within the past week/1–2 weeks ago/2–4 weeks ago/1–3 months ago/more than 3 months ago/never skip medication.” Finally, they were asked questions about their reasons for not taking ART during the last month and perceived side-effects. The same research assistant entered baseline interview dates, out-patient number and the scheduled date for a follow-up interview 6 months later, in both a logbook and the patient's medical file.

All patients, irrespective of treatment duration, were supposed to have a shorter follow-up interview once every 6 months after the baseline questionnaire. Since this was an open cohort including patients who had recently started as well as those who had longer experience of ART, we deemed that a follow-up time of 6 months would allow for every patient to have passed the initial challenging treatment period, and for those still in the program to have gained some routine and experience of the treatment. The follow-up questionnaire focused on self-reported adherence to ART (as measured by a modified AACTG questionnaire [23]). First, the patients were asked the names and doses of each antiretroviral medicine (ARV) they were taking. Drug samples were used to help patients identify their drugs since most patients did not know them by name. Then, patients were asked exactly how many pills they had failed to take over the last four days. Further, patients were asked five adherence questions: “During the past 4 days, on how many days have you missed taking all your doses?”; “Most anti-HIV medications need to be taken on a schedule. How closely have you followed your specific schedule over the last four days?”; “Do your ARVs have special instructions?” followed by “If yes, how often have you followed those special instructions over the last four days?”; “When was the last time you missed taking any of your medications?” and “Did you miss any of your anti-HIV medications last weekend?” (Appendix S1). Patients were finally asked questions about their reasons for not taking ART during the last month and perceived side-effects. The AACTG questionnaire measures adherence during the last four days and during the past weekend [23].

After the first descriptive analysis, the variables in the questionnaires were categorized in different ways for easier interpretation in the logistic models.

In addition to the questionnaires, the dates for clinic appointments and number of prescribed ART doses were collected from individual patient records at the clinic.

### Adherence measures

There is no consensus on what adherence measures to use [24]. Methods include indirect measures (e.g. pill counts, self-reports, electronic monitoring devices and medication refill rates) [10], [25], [26] and direct measures (e.g. observations, drug monitoring and biological markers) [27]. Self-reported measures are quick and inexpensive [28], have been shown to predict clinical outcome [29] and have a significant association with viral load [28]. However, self-reports and pill counts tend to overestimate adherence [29], [30], while medication refill rates need electronic pharmacy data systems in order to be efficient and are not common in sub-Saharan Africa [31]. Adherence indices account for different aspects of adherence [24]. Several other studies have shown that not only dosing, but also the exact timing and compliance with special instructions are important aspects of adherence and impact on viral load [32], [33]. Two separate measures for adherence outcomes were used in this study: dose adherence and an adherence index.

### Dose adherence

Dose adherence was based on data from the follow-up interview on number of doses per drug per day. The number of daily doses was multiplied by four to get the prescribed number of doses over the past four days and then divided by the self-reported number of missed doses on each of the past four days in order to obtain the proportion of prescribed dose actually taken over the past 4 days. At least 95% of a prescribed dose over the last four days was required to be classified as adherent.

### Adherence index

The adherence index was based on questions from the follow-up questionnaire covering dosing, timing and special instructions (Table 1, Appendix S1). An adherence level of 95% was decided for each item: i.e. at least 4/5 for timing, 5/5 for dosing and at least 4/5 for special instructions was required to be classified as adherent. Five out of five was required for dosing since the next step (4/5) meant that patients had missed all doses on one out of five days, i.e. 20% missed dose equal to only 80% adherence. Patients were defined as adherent if they scored ≥13/15 points on the adherence index (Table 1).

**Table 1:** Adherence index: included variables, grading and score.

Adherence aspectCovered	The adherence index by Unge et al	Grading	Score
Timing/Scheduling	‘Most anti-HIV medications need to be taken on a schedule, such as “2 times a day” or “3 times a day” or “every 8 hours.” How closely did you follow your specific schedule over the last four days’	1 Never2 Some of the time3 About half of the time4 Most of the time5 All of the time	4/5
Quantification of adherence	‘During the past 4 days, on how many days have you missed taking all your doses?’	5 None4 One day3 Two days2 Three days1 Four days	5/5
SpecialInstructions	‘How often did you follow those special instructions over the last four days’	1 Never2 Some of the time3 About half of the time4 Most of the time5 All of the time	4/5
		**Maximum score:** **Minimum score to be classified as adherent:**	**15** **≥13/15**

### Drop-out from ART

Drop-out from ART was defined as the patient being without ARVs for at least 90 days. By using routinely collected data from patient records, the number of prescribed daily doses was added to the last clinic appointment date. An additional 90 days were then added to obtain the date when a patient was categorized as an actual drop-out, assuming he or she had not returned before this date. If classified as a drop-out, the date for the last clinic appointment date was used as the drop-out date. A number of patients categorized as drop-outs re-entered the program but kept their classification as drop-outs in the analysis if they had been off ART for more than 3 months, given the high likelihood of viral rebound as well as the increased risk of drug resistance due to suboptimal drug exposure. The patients that did not drop out were censored on the last clinic appointment before the end of the follow-up (20 March 2010).

### Data analysis

Data was entered by the research assistant soon after the performed interviews using the Microsoft Office Access data entry program and exported to SPSS software version 18.0, (SPSS, Inc., Chicago, IL) for statistical analysis. Data was double checked for validity by both the research assistant (AL) and the first author (CU) on several occasions during the study period. Descriptive statistics were collected on socio-demographic characteristics. Mean and standard deviations were computed for numerical variables and proportions for categorical variables. Following the descriptive analysis, bivariate and multivariate logistic regression models were applied to assess the association between patient determinants and the outcome variables; i.e. (1) non-adherence, defined as <95% as measured by the dose adherence, (2) non-adherence, defined as ≤13/15 points to the adherence index and (3) drop-out from the program, defined as not returning more than 90 days after the last given dose of ART.

The association between the two adherence outcomes and baseline data on sex, age, ethnic group, religion, education (primary or secondary school), stable income (employed vs unemployed/casual labour), living below the poverty limit (less than 5 000 KSH/month, about 2 USD/day), number of people in the household, number of biological children, number of other dependants, being a Kibera resident, length of residence in Kibera, disclosed HIV status, having support taking medicines, time to clinic and having been hospitalized since starting ART, was assessed in bivariate analysis. Variables with a p<0.20 were included in the multivariate logistic regression model and removed using a backwards stepwise method (Wald's test). A value of p<0.05 was considered statistically significant in the final models. Odds ratios (ORs) were always adjusted for age and sex regardless of p-value. The final goodness of fit of the model was tested using Hosmer-Lemeshow. A survival analysis (Cox regression model with proportional hazard assumption tested with Shoenfeld residuals) was also performed using the retrospective dataset, to calculate the hazard ratios for the drop-out, their p-value and 95% confidence intervals (CI). The survival analysis also included a graphic presentation using a survival curve (time in days on the X axis and survival cumulative function in Y axis) with ART initiation as time zero and the event “not returning for more than 90 days after last given dose” as the loss to clinic appointment date.

### Ethical Standards

All the 800 ART clients who were asked to participate agreed to be interviewed, following written informed consent. Ethical approval was obtained from the Kenya Medical Research Institute (KEMRI) and the Regional Ethical Review Board in Stockholm, Sweden.

## Results

### Basic characteristics

Demographic data are shown in Table 2, 3, 4. A total of 800 patients (mean age 37 years, 66% females) were included in the baseline assessment. The average household consisted of a widow who resided with 2–3 people excluding herself, having 2–3 biological children and supporting 6 people, sometimes outside the household. Seventy-five percent of the patients were living in Kibera, two-thirds for more than 5 years. Less than half the patients (49%) had known their HIV status for over 2 years but most (83%) had disclosed to someone. The mean time on ART was 23 (2–53) months. Only 40% of the respondents had a formal treatment buddy while 50% had friends or family members helping them to remember their medicines. A majority (60%) were satisfied with the support they received from family and friends. Thirty percent used alcohol, 8% consumed at least one unit of alcohol per day, while the majority, 70%, said they never consumed alcohol and only 0.6% admitted to ever using any social drugs (heroin, marijuana, cocaine, khat or kuber).

**Table 2:** Characteristics of the ART patients included in the cohort study (N = 800).

Characteristics		n (%)	Missing
Sex			3
	Males	274 (34.4)	
	Females	523 (65.6)	
Mean age ±SD		37.2±8.6	3
Mean age by sex	Males	40.6	
	Females	35.8	
Mean time on ART±SD		23.0±20.2	188
Ethnic group			3
	Lou	251 (31.5)	
	Kisii	39 (4.9)	
	Kamba	163 (20.5)	
	Kikuyu	86 (10.8)	
	Maasai	3 (0.4)	
	Luhya	217 (27.2)	
	Nubien	12 (1.5)	
	Other	26 (3.3)	
Religion			12
	Protestant	476 (60.4)	
	Catholic	253 (32.1)	
	Muslim	23 (2.9)	
	Other	36 (4.6)	
Highest education achieved			12
	Up to primary school	473 (60.0)	
	Secondary school/higher	315 (39.4)	
Present occupation			25
	Non stable income	390 (50.3)	
	Stable income	385 (49.7)	
Income/month			218
	<5000 KSH	236 (40.5)	
	>5000 KSH	346 (59.5)	
Relationship status			12
	One partner	385 (48.9)	
	Two partners or more	26 (3.3)	
	Widow/widower	155 (19.7)	
	Single	119 (15.1)	
	Divorced/separated	103 (13.1)	
Number of people in household			12
	0	92 (11.7)	
	1	97 (12.3)	
	2–3	274 (34.8)	
	>4	325 (41.2)	

**Table 3:** Table 2 continued.

Characteristics		n (%)	Missing (n)
Number of children			12
	0	75 (9.5)	
	1	131 (16.6)	
	2–3	338 (42.9)	
	>4	244 (30.9)	
Number of people supporting financially			13
	0	141 (17.9)	
	1	61 (7.8)	
	2–3	178 (22.6)	
	4–5	184 (23.4)	
	>6	223 (28.3)	
Living in Kibera			12
	No	201 (25.5)	
	Yes	587 (74.5)	
Time living in Kibera			213
	0–2 years	77 (13.1)	
	2–5 years	139 (23.7)	
	>5 years	371 (63.2)	
Time to reach clinic from residence			12
	Less than 10 minutes	74 (9.4)	
	10–30 minutes	431 (54.7)	
	31–60 minutes	174 (22.1)	
	More than one hour	109 (13.8)	
Time since HIV diagnosis			12
	<6 months ago	89 (11.3)	
	6–12 months ago	121 (15.4)	
	1–2 years ago	195 (24.7)	
	over 2 years ago	383 (48.6)	
Disclosed status to anyone			12
	Yes	653 (82.9)	
	No	135 (17.1)	
Initial ART provider			190
	AMREF	535 (87.7)	
	MSF	7 (1.1)	
	Private vendor	13 (2.1)	
	Other	55 (9.0)	
Have a treatment buddy			12
	Yes	315 (40.0)	
	No	473 (60.0)	
Satisfied with support from friends/family			12
	Yes	472 (59.9)	
	No	316 (40.1)	

**Table 4:** Antiretroviral drugs used at last follow up, AMREF clinic (N = 1770).

Antiretroviral drugs used at last follow up		ART	n (%)
	First line treatment		
		Stavudine	649 (36.7)
		Lamivudine	1530 (97.6)
		Nevirapine/Efvavirenz	1517 (85.7)
	Second line treatment		
		Zidovudine	619 (35.0)
		Didanosine	17 (1.0)
		Lopinavir	42 (2.7)
		Zidovudine	619 (35.0)

Out of the 800 patients interviewed at baseline, 352 were included in at least one follow-up interview. Patients were followed up for a total period of 1 828 person-years.

### Adherence outcomes

Results from the bivariate analysis are presented in Table 5 and 6.

**Table 5:** Bivariate analysis of background factors with respect to dose adherence and adherence index (N = 352).

		Dose adherent		Adherent index	
		Adherent	Non adherent	Adherent	Non adherent
		n (%)	n (%)	n (%)	n (%)
Sex	Females	163 (63.2)	27 (81.8)	140 65.4)	91 (70.0)
	Males	95 (36.8)	6 (18.2)	74 (34.6)	39 (30.0)
Mean age±SD		37.5±8.3	37.4±7.2	37.9±8.5	37.0±8.2
Ethnic group	Lou	88 (34.1)	8 (24.2)	73 (34.1)	38 (29.2)
	Kisii	11 (4.3)	0 (0.0)	6 (2.8)	10 (7.7)
	Kamba	50 (19.4)	6 (18.2)	52 (24.3)	22 (16.9)
	Kikuyu	24 (9.3)	7 (21.2)	17 (7.9)	16 (12.3)
	Maasai	1 (0.4)	0 (0.0)	1 (0.5)	0 (0.0)
	Luhya	72 (27.9)	10 (30.3)	52 (24.3)	36 (27.7)
	Nubien	5 (1.9)	1 (3.0)	5 (2.3)	2 (1.5)
	Other	7 (2.7)	1 (3.0)	8 (3.7)	6 (4.6)
Religion	Protestant	145 (57.5)	17 (51.5)	115 (55.3)	71 (55.9)
	Catholic	79 (31.3)	11 (33.3)	70 (33.7)	40 (31.5)
	Muslim	8 (3.2)	1 (3.0)	10 (4.8)	3 (2.4)
	Other	20 (7.9)	4 (12.1)	13 (6.3)	13 (10.2)
Highest education	Up to primary school	137 (54.4)	26 (78.8)	108 (51.9)	86 (67.7)
	Secondary school/higher	115 (45.6)	7 (21.2)	100 (48.1)	41 (32.3)
Present occupation	Non stable income	123 (49.4)	14 (42.4)	98 (47.8)	76 (59.8)
	Stable income	126 (50.6)	19 (57.6)	107 (52.2)	51 (40.2)
Poverty	<5000 KSH/month	57 (30.3)	14 (58.3)	51 (32.5)	33 (37.1)
	>5000 KSH/month	131 (69.7)	10 (41.7)	106 (67.5)	56 (62.9)
Marital status	One partner	121 (48.0)	12 (36.4)	96 (46.2)	58 (45.7)
	>One partner	6 (2.4)	2 (6.1)	5 (2.4)	6 (4.7)
	Widow/widower	51 (20.2)	11 (33.3)	46 (22.1)	30 (23.6)
	Single	36 (14.3)	5 (15.2)	31 (14.9)	17 (13.4)
	Divorced/separated	38 (15.1)	3 (9.1)	30 (14.4)	16 (12.6)
People residing with	0	32 (12.7)	7 (21.2)	96 (46.2)	58 (45.7)
	1	30 (11.9)	0 (0.0)	5 (2.4)	6 (4.7)
	2–3	72 (28.6)	10 (30.3)	46 (22.1)	30 (23.6)
	4–5	76 (30.2)	10 (30.3)	31 (14.9)	17 (13.4)
	over 6	42 (16.7)	6 (18.2)	30 (14.4)	16 (12.6)
Number of children	0	22 (8.7)	1 (3.0)	16 (7.7)	9 (7.1)
	1	43 (17.1)	4 (12.1)	36 (17.3)	18 (14.2)
	2–3	109 (43.3)	18 (54.5)	89 (42.8)	55 (43.3)
	4–5	53 (21.0)	6 (18.2)	45 (21.6)	28 (22.0)
	6–7	19 (7.5)	2 (6.1)	18 (8.7)	11 (8.7)
	over 8	6 (2.4)	2 (6.1)	4 (1.9)	6 (4.7)

**Table 6:** Table 5 continued.

		Dose adherent		Adherent index	
		Adherent	Non adherent	Adherent	Non adherent
		n (%)	n (%)	n (%)	n (%)
People supporting financially	0	39 (15.5)	5 (15.2)	34 (16.3)	20 (15.7)
	1	14 (5.6)	1 (3.0)	11 (5.3)	10 (7.9)
	2–3	61 (24.2)	7 (21.2)	49 (23.6)	28 (22.0)
	4–5	61 (24.2)	9 (27.3)	53 (25.5)	21 (16.5)
	6–7	37 (14)	6 (18.2)	27 (13.0)	27 (21.3)
	over 8	40 (15.9)	5 (15.2)	34 (16.3)	21 (16.5)
Living in Kibera		185 (73.4)	24 (72.7)	151 (72.6)	97 (76.4)
Time living in Kibera	0–2 years	20 (10.8)	3 (12.5)	14 (9.3)	11 (11.3)
	2–5 years	35 (18.9)	5 (20.8)	33 (21.9)	15 (15.5)
	over 5 years	130 (70.3)	16 (66.7)	104 (68.9)	71 (73.2)
Time to reach clinic	Less than 10 minutes	12 (4.8)	4 (12.1)	10 (4.8)	9 (7.1)
	10–30 minutes	146 (57.9)	17 (51.5)	122 (58.7)	68 (53.5)
	31–60 minutes	62 (24.6)	9 (27.3)	49 (23.6)	37 (29.1)
	More than one hour	32 (12.7)	3 (9.1)	27 (13.0)	13 (10.2)
Learned about HIV status	<6 months ago	28 (11.1)	9 (27.3)	22 (10.6)	16 (12.6)
	6–12 months ago	31 (12.3)	6 (18.2)	26 (12.5)	15 (11.8)
	1–2 years ago	67 (26.6)	5 (15.2)	50 (24.0)	33 (26.0)
	over 2 years ago	126 (50.0)	22 (66.7)	188 (90.4)	105 (82.7)
Disclosed status to anyone		225 (89.3)	22 (66.7)	188 (90.4)	105 (82.7)
Got the ARV from in the beginning	AMREF	222 (88.1)	31 (93.9)	183 (88.0)	102 (87.2)
	MSF	4 (1.6)	0 (0.0)	4 (1.9)	0 (0.0)
	Private vendor	2 (0.8)	0 (0.0)	0 (0.0)	1 (0.9)
	Other	24 (9.5)	2 (6.1)	21 (10.1)	14 (12.0)
Been hospitalized after starting ARV		33 (13.1)	6 (18.2)	28 (13.5)	17 (14.5)
Have a treatment buddy		106 (42.1)	13 (39.4)	99 (47.6)	45 (35.4)
Satisfied with support from friends and family		165 (65.5)	21 (63.6)	135 (64.9)	80 (63.0)
Friends or family members help remember		130 (51.6)	13 (39.4)	113 (54.3)	56 (44.1)
Mean time on ARV±SD		23.1±19.2	14.4±12.7	23.5±18.5	23.5±21.1

Using dose adherence based on the number of missed doses over the last four days, 11% (n = 33) of the patients were non-adherent (<95%) at six-month follow-up. The following variables were significantly associated with non-adherence in bivariate analysis: sex (female), undisclosed HIV status, not satisfied with support in taking ART medicines, low level of education, living below poverty limit (<US$ 2/day), short distance to clinic and shorter average time on ART (non-adherent = 14.4 months on ARV; adherent = 23.1 months on ARV). In the final multivariate analysis, undisclosed HIV status (OR 4.70, 95% CI = 1.78–12.43) and living below the poverty limit (OR 3.28, 95% CI = 1.27–8.48) remained significantly associated with dose adherence <95%, adjusting for age and sex.

**Figure 1:**
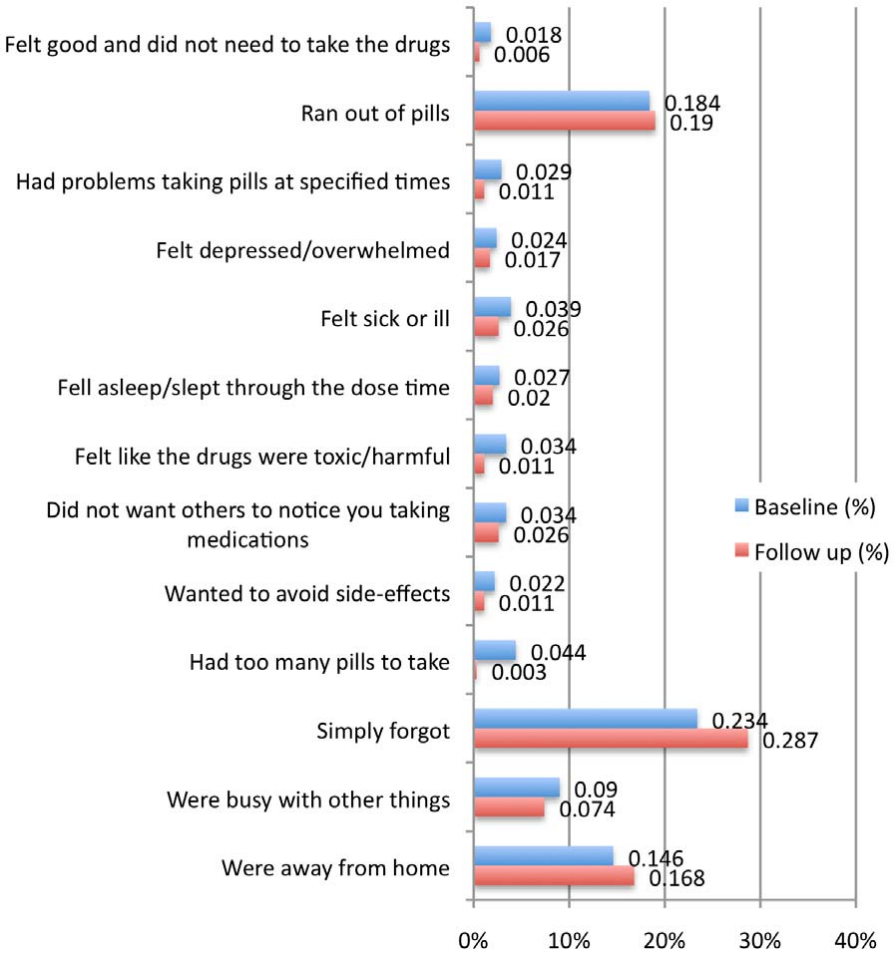
Reasons for not taking ART during the last month at baseline and follow up.

When asked to report long-term adherence, 37% of the 352 patients said they had missed at least one of their ARV doses at some time between the past week and the last 3 months and as many as 15% had missed taking their ART the previous weekend (Appendix S1). The most common reasons stated for missing drugs over the past month were “simply forgot” (28%) and “ran out of pills” (19%). Other reasons for missing drugs at baseline and follow-up are summarized in Figure 1.

**Figure 2:**
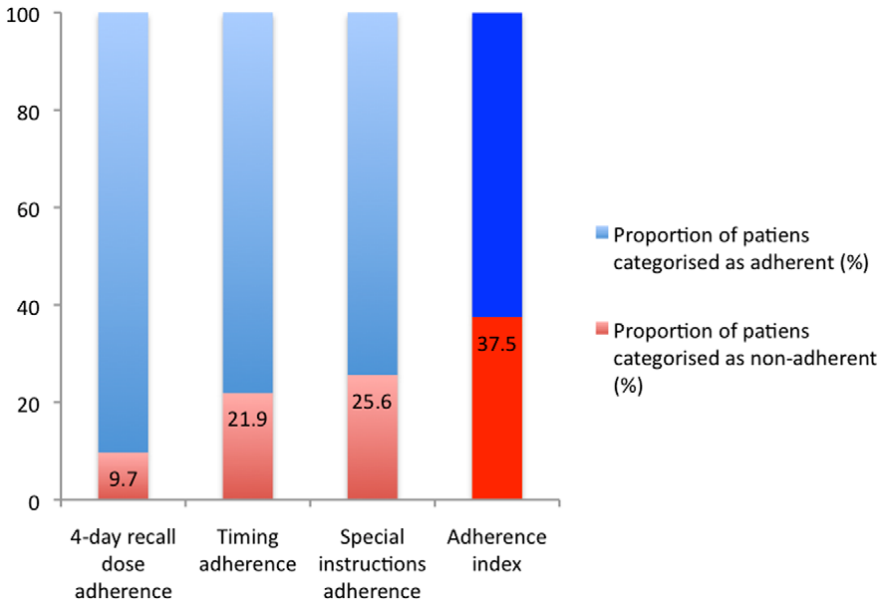
Different aspects of self-reported adherence at 6 months follow-up: 4-day recall dosing, timing, special instructions, and adherence index including all three aspects. **N = 352**.

According to the adherence index based on dose, timing and capacity to follow special drug instructions, 38% (130) patients were classified as non-adherent (Figure 2). The following variables were significantly associated with a low adherence index in bivariate analyses: undisclosed HIV status, not satisfied with support in taking ART, not having a treatment buddy, low level of education and unstable income. The following variables remained significantly associated with a low adherence index in the final multivariate analysis: not having a treatment buddy (OR 1.60, 95% CI = 1.01–2.54) and low education (OR 1.95, 95% CI = 1.21–3.15) also adjusted for age and sex.

### Drop-out from program

**Figure 3:**
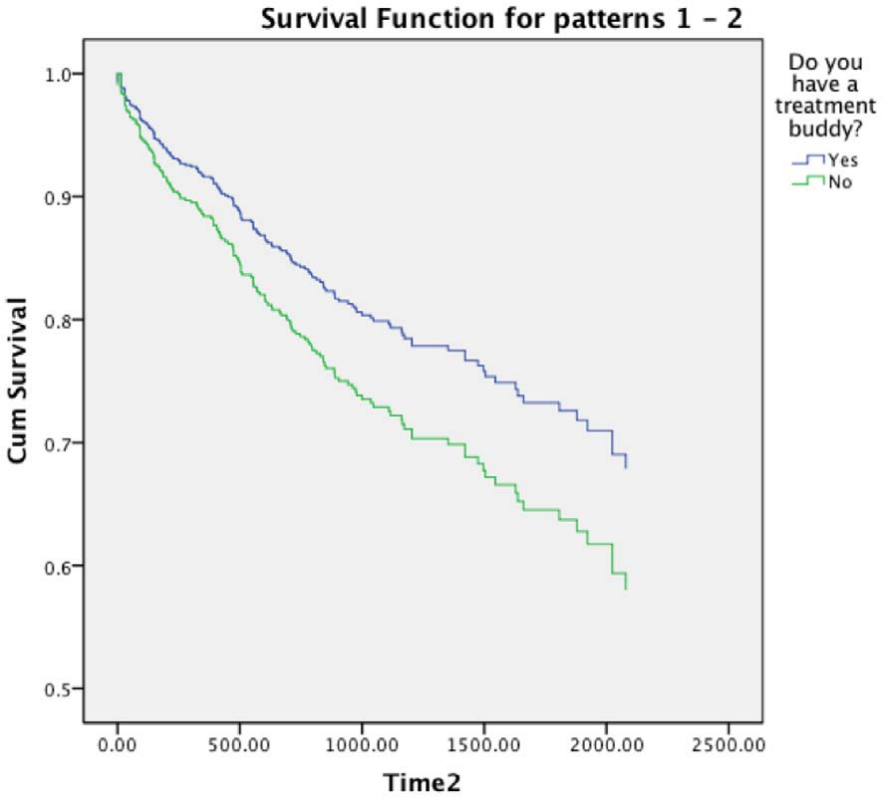
Survival function (days) for patients according to Treatment buddy.

Of the 800 patients on ART included in the baseline assessment, 101 patients were excluded from the survival analysis due to missing data on appointment dates and number of doses prescribed. Of the 699 patients included in the analysis, 163 (23%) dropped out for more than 90 days after the last prescribed dose, leaving 536 (77%) in the ART program at the end of the study. The total number of clinic appointment years of follow-up was 1 828. The Cox regression model showed a significantly higher hazard ratio for people not having a treatment buddy (HR 1.41, 95% CI = 1.02–1.94), adjusted for age and sex (Figure 3).

### Missing data

Data on clinic appointment dates and number of prescribed doses were missing for 101 patients mainly due to inconsistencies in outpatient numbers and/or clinic appointment dates. Further, information on what happened to the drop-outs was not available although efforts were made to trace these drop-outs with the help of community health workers. A few variables included in the statistical modelling (time in Kibera, time since ART initiation, income level, having had another previous ART provider or being hospitalized due to AIDS) had a substantial amount of missing values (Table 2) and further analyses were therefore performed to assess the potential of non-random bias: The great majority, 94% (201/213), of those with missing data on time living in Kibera were non-Kibera residents. Patients with missing data on time on ART did not differ significantly in terms of gender, age or ethnicity compared to other patients in the study, indicating a random distribution of missing values. In this population time since HIV diagnosis closely coincides with time since ART initiation to a large extent making up for the missing values on time on ART. Among those with missing information on income level (< or >5000 Ksh), 91% (198/218) reported that they lacked a stable and predictable income. Since AMREF was the first provider of ART in Kibera it is unlikely that patients included in this long-term follow-up would have been enrolled in another ART program previous to our assessment. Lastly, any substantial degree of hospitalization due to AIDS-related symptoms is unlikely in this population given the lack of tertiary level care in Kibera. The hypothesis that our data was missing completely at random (MCAR) was also statistically verified using Little's MCAR test (p-value equal to 0.259) rejecting any systemic bias in terms of missing data. In conclusion, the likelihood is low that the missing values could have biased our results away from the null hypothesis.

## Discussion

In this prospective cohort study of 800 HIV patients on ART in the Kibera slum, Nairobi, Kenya, over a period of 2.5 years, more than a third of patients were non-adherent taking dose, timing and special instructions for ARVs into account. In addition, one in four dropped out more than 90 days after their last prescribed ART dose. Living below the poverty limit, having a low education and lacking family support (non-disclosure or not having a formal treatment buddy) were significant risk factors for non-adherence. This raises urgent questions about the difficulties associated with sustaining a growing number of individuals on life-long ART, while actual investments in poverty and stigma reduction and in schooling are seriously lagging behind in urban slums.

Eleven percent of the patients in this cohort were non-adherent according to the dose adherence calculations based on the self-reported number of pills missed during the last four days. These findings are consistent with other studies in sub-Saharan Africa [34]. In most adherence studies, the percentage of missed doses during the last three or four days is usually the only aspect of adherence being assessed [21]. Since many ART regimens demand that patients take their pills at exact times, and that some ART must be taken with food or on an empty stomach, adherence is more than just taking the right number of pills per day [32], [33], [35], [36]. Therefore, an adherence index was created, taking into account timing and special instructions, in addition to dose adherence.

The fact that the proportion of patients classified as non-adherent increased from 11% to 38% depending on the type of adherence measure used (i.e. dose adherence versus adherence index) indicates that patients often are classified as being adherent when only the number of missed pills during the last few days are assessed. Thus, it is important to view adherence as multi-factorial and to assess different aspects including dose timing and capacity to follow food restrictions, in order to achieve virologic suppression [32], [33]. However, the importance of following exact schedules and food instructions is dependant on type of drug combination and of special concern for patients on protease inhibitors (PIs) [37]. Although in the present cohort only 0.8% of the patients were on a PI-based regimen, also some nucleoside reverse transcriptase inhibitors (NRTIs) (e.g. didanosine) and non-nucleoside reverse transcriptase inhibitors (NNRTIs) (e.g. efavirenz), both frequently used antiretrovirals at the AMREF clinic, are associated with food restrictions [38]. In this cohort, 38% of the patients had a low adherence index score assessing timing and special instructions in addition to dosing. This is in line with earlier studies from other resource-poor African settings where 47–78% of patients are categorized as adherent when dosing, timing and food instructions are taken into account [32], [34], [35].

The most common reason for missing pills, simply forgetting to take them, could be abridged through different intervention strategies for reminding patients to take their HIV medication such as electronic or telephone-based alarms and home visits [39], [40]. The second most common reason reported by patients for not taking their drugs was “ran out of pills”. The AMREF clinic did not suffer from stock-outs of drugs which otherwise is a serious barrier to high-quality ART programmes especially for government providers in rural sub-Saharan Africa [41], but patients coming late for drug refill was a frequent problem at the clinic. The staff, also identified patients' ignorance of the importance of avoiding treatment interruption, refusal to come for drug pick up, or travels up country to visit relatives without informing their doctor, as other possible reasons for running out of ARVs.

Following food instructions is especially difficult in resource-poor settings where food insecurity is widespread. The fact that about 25% of the patients in this cohort did not adhere to special instructions (Figure 2), including food, could be interpreted from a poverty perspective: 50% were unemployed or had unstable incomes due to casual labor, 41% were classified as living in absolute poverty. On top of this, an average Kibera citizen financially supports 4–5 children and lives under dismal conditions making it extremely difficult to follow special food instructions for ART, as shown in a previous qualitative study in the same setting [42], and indicated by other research [19], [43], [44].

Ensuring free access to ART and reducing transports costs are often-mentioned initial interventions that promote adherence at community and individual level [45], [46], but for patients in the Kibera slum, where ART is free of charge and people live at close walking distance from clinics, stigma and lack of social support have previously been shown to be more important barriers to ART initiation [42]. Not disclosing HIV status and lack of a formal treatment buddy were found to be significant, independent, predictors for non-adherence in line with previous research [20], [34], [45], [47], [48]. Although disclosure in order to attain social support is desirable, stigma against people living with HIV is still widespread according to a recent qualitative study performed by our team in Kibera (unpublished results), and difficulties for patients on ART to choose when and to whom they would like to disclose, in an over-crowded context like the Kibera slum, is an important reason for dropping out of ART [18], [42].

Based on the clinic appointment dates and number of prescribed doses, 163 (23%) of the patients dropped out of the ART program for more than 90 days after the last prescribed dose. In this study drop-out was used synonymously with loss to follow-up (LTFU). Other studies from sub-Saharan Africa suggest that much of LFTU is due to self-transfer between ART programs [49], [50], [51]. Sustaining ART in urban slums is an increasing challenge given the growing number of ART providers including NGOs and bilateral donors, some of them competing openly for HIV patients in Kibera with no coordinated referral between programs. Several clinics for example provide food packages for HIV-infected patients to attract other people short of food and the provision of baby formula is one of the most attractive incentives for young pregnant women living with HIV to self-transfer from the AMREF clinic to other providers. In the present study data were not available as to where the patients being classified as drop-outs had gone but the clinic staff indicated that many probably had self-transferred to nearby facilities that offered more benefits such as food packages etc. Thus, although program loss is not necessarily equal to treatment loss for the individual patient, it is still associated with a considerable risk of at least temporary treatment interruption. A Ugandan study found that 83% of patients initially classified as LTFU could be traced to another ART program [52], but the extent to which treatment interruptions due to self-transfer or unguided program switch had clinical or virological drawbacks was not studied. Underlying individual circumstances for not having a treatment buddy (found to be significantly associated with drop-out in our survival analysis) are often strongly influenced by stigma and lack of social support, all unlikely to change, at least in the short-term, regardless of ART provider.

The strength of this study was that it was unique in its kind, conducted in an urban slum, a very complex study area and home to around one million highly mobile multi-ethnic people, living in an area the size of Central Park in New York. In the very near future most patients in need of ART in the world will live in similar environments. Logistic challenges including security to perform studies in this environment are enormous, yet interesting findings were revealed in collaboration with the staff at the clinic. The prospective study design enabled us to retrieve more accurate follow-up information on adherence that could be linked to baseline data, and also enabled the interviewer to validate patient drop-outs directly with the staff while these patients were still fresh in memory.

One limitation of the study was the amount of missing data, caused by a number of issues, but following further analyses judged to be random in nature. The fact that we studied adherence among patients retained in care, but lacked adherence data on patients LTFU, may have introduced a potential systematic bias underestimating the true risk of low adherence since patients that remain in the programme are more likely to be adherent. Due to economic and logistical restraints, the relationship between adherence outcomes and CD4 counts or viral load was not possible to assess. The <95% threshold used for classification of non-adherence may have been too stringent. This often cited measure comes from studies performed with unboosted PIs [10], but later studies have shown that viral suppression may be achieved with adherence levels of 50%–80% based on NNRTIs [9].

Since 70% of urban populations in sub-Saharan Africa live in slums, sustaining HIV patients on ART in these high-risk and highly mobile settings is a major future challenge. The high proportion of patients dropping out of treatment programs and being non-adherent must be addressed using context-specific solutions like extended counselling and community-based treatment support. If policymakers and funders are serious about making life-long ART available for patients in sub-Saharan Africa, it is important to reduce competition between providers and avoid the short-term funding strategies seen in this area. It is equally important to invest in poverty reduction strategies and education.

## Supporting Information

Appendix S1 Questions from the follow-up questionnaire.
(0.03 MB DOC)
