# Supplementary material for: Long-Term Adherence to Antiretroviral Treatment and Program Drop-Out in a High-Risk Urban Setting in Sub-Saharan Africa: A Prospective Cohort Study
Source: PLoS One. 2010 Oct 25;5(10):e13613. doi: 10.1371/journal.pone.0013613 (PMC2963610; doi:10.1371/journal.pone.0013613)
Supplement: Appendix S1 — Questions from the follow-up questionnaire. (0.03 MB DOC) [file pone.0013613.s001.doc]

**APPENDIX S1**

**Questions from the follow-up questionnaire.**

**___________________________________________________________________________**

Follow-up question Value (%)

”During the past 4 days, on how many days have you missed taking all your doses?”

None 318 (90.3)

One day 23 (6.5)

Two days 4 (1.1)

Three days 5 (1.4)

Four days 2 (0.6)

”Most anti-HIV medications need to be taken on a schedule. How closely did you follow your specific schedule over the last four days?”

Never 7 (2.0)

Some of the time 62 (17.6)

About half of the time 8 (2.3)

Most of the time 120 (34.1)

All of the time 155 (44.0)

“Do your ARVs have special instructions?”

Yes 347 (98.6)

“If yes, how often did you follow those special instructions over the last four days?”

Never 14 (4.0)

Some of the time 63 (18.2)

About half of the time 12 (3.5)

Most of the time 69 (19.9)

All of the time 189 (54.5)

___________________________________________________________________________

Additional adherence questions from follow up questionnaire:

___________________________________________________________________________

“When was the last time you missed taking any of your medications?”

Within the past week 53 (15.1)

1-2 weeks ago 11 (3.1)

2-4 weeks ago 6 (1.7)

1-3 months ago 33 (9.4)

> 3 months ago 27 (7.7)

Never 222 (63.1)

“Did you miss any of your anti-HIV medications last weekend?”

Yes 53 (15.1)
